# Component processes in free-viewing visual search: Insights from fixation-aligned pupillary response averaging

**DOI:** 10.1167/jov.20.7.5

**Published:** 2020-07-07

**Authors:** Joel T. Martin, Annalise H. Whittaker, Stephen J. Johnston

**Affiliations:** 1 School of Human and Health Sciences, Department of Psychology, University of Swansea, Swansea, Wales, UK; 2 Institute of Biomedical Engineering, University of Oxford, UK; 3 Defence Science and Technology Laboratory (DSTL), Porton Down, Salisbury, UK

**Keywords:** visual search, pupillometry, target detection, visual attention, locus coeruleus, noradrenalin

## Abstract

Pupil size changes during a visual search may reflect cognitive processes, such as effort and memory accumulation, but methodological confounds and the general lack of literature in this area leave the reliability of findings open to question. We used a novel synthesis of experimental methods and averaging techniques to explore how cognitive processing unfolds during free-viewing visual search for multiple targets. Twenty-seven participants completed 152 searches across two separate 1-hour sessions. The number of targets present (Targets: 0, 1, 2, and 3) in each trial was the main manipulation and the task was to “find all of the targets” and report the total via mouse-click at the end of the trial. Search time lasted for 10 seconds or until the participant purported to have found all of the targets, in which case they could terminate the search via keypress. Whole-trial pupil analysis revealed a significant effect of button pressing as well as a significant main effect of targets for trials that were not self-terminated via button press. Fixation-aligned pupil responses revealed transient modulations in pupil size following initial fixations on targets but not distractors and refixations on both targets and distractors. Owing to rigorous control over experimental confounds and a detailed analysis and correction of eye-movement-related measurement error, we confidently discuss these findings in terms of task-related processing and underlying brain activity.

## Introduction

Pupillometry, the measurement of pupil size, has become a popular tool for researching cognitive function. Its utility for this purpose hinges to a large extent on our growing understanding of the functional association between non-luminance-mediated pupil size changes and the activity of the locus coeruleus-noradrenalin (LC-NA) system of the brain, which plays a pivotal role in the modulation of arousal, cognition, and autonomic function (Berridge, 2008; Berridge & Waterhouse, 2003; Doya, 2008; Mather & Harley, 2016; Samuels & Szabadi, 2008a, Samuels & Szabadi, 2008b; Sara, 2009; Sara & Bouret, 2012). The most direct and striking evidence for a functional pupil-locus coeruleus (LC) link comes from neural recording and microstimulation studies in monkeys, which have revealed a strong temporal coupling between LC activity and non-luminance-mediated changes in pupil size (Joshi, Li, Kalwani, & Gold, 2016; Rajkowski, Kubiak, & Aston-Jones, 1993; Varazzani, San-Galli, Gilardeau, & Bouret, 2015), but further evidence of a less-direct nature comes from a broad range of human studies, which have combined pupil size measurements with pharmacological manipulations, functional imaging, and experimental manipulation of common factors known to drive changes in pupil size and LC activity (Alnaes et al., 2014; Aston-Jones & Cohen, 2005; Beatty, 1982a, Beatty, 1982b; de Gee et al., 2017; Einhäuser, Stout, Koch, & Carter, 2008; Gilzenrat, Nieuwenhuis, Jepma, & Cohen, 2010; Hou, Freeman, Langley, Szabadi, & Bradshaw, 2005; Jepma & Nieuwenhuis, 2011; Morad, Lemberg, Yofe, & Dagan, 2000; Murphy, O'Connell, O'Sullivan, Robertson, & Balsters, 2014; Murphy, Robertson, Balsters, & O'Connell, 2011; Phillips, Szabadi, & Bradshaw, 2000; Richer & Beatty, 1987; Urai, Braun, & Donner, 2017). Overall, the evidence strongly suggests that pupil dynamics under conditions of constant luminance serve as a reliable proxy for the moment-to-moment activity of the LC, and by extension as a basis of inference for the cognitive processes that are associated with this otherwise elusive subcortical nucleus.

To date, pupillometry has been used to study arousal and interest (e.g. Aboyoun & Dabbs, 1998; Hess & Polt, 1960; Hess, Seltzer, & Shlien, 1965), cognitive load (e.g. Ahern & Beatty, 1979, Ahern & Beatty, 1981; Hess & Polt, 1964; Kahneman & Beatty, 1966), memory (e.g. Magliero, 1983; Võ et al., 2008), attentional orienting and salience (e.g. Lynn, 1966; Wang & Munoz, 2014), decision making (e.g. Cavanagh, Wiecki, Kochar, & Frank, 2014; Einhäuser, Koch, & Carter, 2010; Simpson & Hale, 1969), and many other aspects of cognition in a broad range of well-established paradigms. But despite its growing popularity and widespread use in experimental psychology, there have been few pupillometric studies of visual search. Visual search is a task with which most of us are familiar, whether it is in the everyday sense of looking for faces in crowds, keys on cluttered surfaces, cars in crowded car parks; or in the laboratory sense of looking for target items in arrays of nontarget items. The lack of pupillometry studies in the otherwise burgeoning field of visual search is perhaps unsurprising when we consider the methodological complications associated with visual stimuli and with eye-movement-related pupil measurement error inherent to video-based systems. Regarding the first of these complications, it is well-known that the pupil responds to low-level stimulus characteristics, such as color, spatial frequency, contrast, size, movement, and, most notably, luminance (Barbur, Harlow, & Sahraie, 1992; Barbur, Wolf, & Lennie, 1998; Goldwater, 1972; Kohn & Clynes, 1969; Slooter & van Norren, 1980; Ukai, 1985; Watson & Yellott, 2012; Woodhouse, 1975). This limits the range of stimuli that can be used effectively to isolate cognitive pupillary responses and often leaves room for doubt as to whether experimental effects were brought about by the psychological rather than the physical aspects of the stimuli. The second issue, more technical in nature, is born out of the fact that video-based eye trackers often derive pupil size measurements from the two-dimensional image of the pupil that gets projected onto a camera sensor. The camera of course views the eye from a fixed position, so eye rotation causes the image of the pupil to become distorted and, in turn, leads to measurement error. This effect, hereafter referred to as the pupil foreshortening error (PFE; Hayes & Petrov, 2015), should be circumvented through experimental design (e.g. do not allow eye movements during trials) or remedied with some form of corrective procedure (Brisson et al., 2013; Gagl, Hawelka, & Hutzler, 2011; Hayes & Petrov, 2015; Pomplun & Sunkara, 2003). As visual search naturally involves visual stimuli and eye movements, the combination of these issues presents a complex methodological challenge for pupillometry.

Notwithstanding the aforementioned complications, there have been a handful of well-executed and informative pupillometric studies of visual search. Perhaps the first in this regard were Porter, Troscianko, and Gilchrist (2007), who explored how patterns of pupil dilation relate to the variable effort required for search tasks of different difficulty. In the first of two experiments, pupil dilation was greater for harder present-absent searches (i.e. those with a larger set size and / or heterogeneous distractors), but it was greater still in a control condition where the task was to report whether the total number of items was odd or even. The difference in dilation between the search and counting tasks appeared to be related to the differential load they placed on locational memory: accuracy in counting required perfect locational memory from the outset to avoid missing or double-counting an item, whereas in the present-absent search for targets, accuracy did not depend so obviously on locational memory. This suggested that pupil dilation in visual search was indicative of spatial memory load, an interpretation that resonates with the literature implicating pupil dilation in memory load (Kahneman & Beatty, 1966; Klingner, Tversky, & Hanrahan, 2011; Piquado, Isaacowitz, & Wingfield, 2010; Van Gerven, Paas, Van Merriënboer, & Schmidt, 2004) and memory in search (Gibson, Li, Skow, Brown, & Cooke, 2000; Gilchrist & Harvey, 2000; Kristjánsson, 2000; McCarley, Wang, Kramer, Irwin, & Peterson, 2003; Petersen, Kramer, Wang, Irwin, & McCarley, 2001; Shen, McIntosh, & Ryan, 2014; Shore & Klein, 2000). The second experiment, requiring search for multiple targets, gave credence to this interpretation. In two separate conditions, participants reported (a) whether a target was present or absent, and (b) whether one or two targets were present. In this scenario, dilation followed a similar pattern across the phase of performance, but it was accelerated slightly in a window toward the end of the search in the one- or two-target condition, again suggesting that pupil dilation may be reflecting the accumulation of memory for previously visited locations.

The experiments of Porter, Troscianko, and Gilchrist (2007) showed that valuable insights into visual search cognition might be gleaned from pupillometry, but there are a number of caveats that warn against a strict effort or memory interpretation of their findings. For example, we know that the cognitive pupillary response reflects the superposition of all cognitive processes that are currently active insofar as they modulate the tone of the autonomic nervous system (Loewenfeld, 1993). This is a broad range of cognitive processes (for reviews, see: Andreassi, 2000; Beatty, 1982b; Goldwater, 1972; Laeng, Sirois, & Gredeback, 2012; Peinkhofer, Knudsen, Moretti, & Kondziella, 2019), and the analysis techniques used by Porter et al. (2007) focusing as they did on the pattern of pupil dilation across the whole trial, were not optimized to differentiate between them. Further, even when luminance is controlled for as rigorously as it was by Porter et al., there are specific task components in visual search, unrelated to effort or memory, which are known to contribute substantially to pupil dilation. Visual target detection (Klingner, 2010a; Privitera, Renninger, Carney, Klein, & Aguilar, 2010; Wierda, van Rijn, Taatgen, & Martens, 2012) and motor response preparation and execution (Hupé, Lamirel, & Lorenceau, 2009; Kloosterman et al., 2015; Privitera, Carney, Klein, & Aguilar, 2014; Privitera et al., 2010; Richer & Beatty, 1985) are two notable examples, both of which were present in Porter et al.'s experiments. Finally, and on a more technical note, Porter et al. made recordings with a locally developed head-mounted video-based system, which measured the horizontal width of the pupil, always at its widest point (Alexandridis, Leendertz, & Barbur, 1991). This implies that the optical distortion accompanying horizontal eye movements was a source of measurement error, an issue not discussed by the authors. Could participants have adopted common eye movement strategies (e.g. searching or counting from left to right), thereby introducing a systematic PFE effect? This seems plausible given the nature of the tasks and their instructions, but it cannot be verified as the system did not record eye movements.

Another valuable study, conducted by Klingner (2010a), evaluated the efficacy of a novel technique called *fixation-aligned pupillary response averaging* for isolating components of the pupil response in visual search. The technique uses gaze data to identify subtask epochs that occur at unpredictable times during a visual task. Once identified, the epochs are temporally aligned and averaged together, with the resulting waveforms used to make inferences about the cognitive processing, which accompanied the events of interest. An advantage of this approach over the “whole trial” approach used by Porter et al. is that epochs can be well-separated in time from other noncognitive events known to cause pupil dilation, such as display changes and button-press responses, allowing for greater specificity at inference. Klingner (2010a) demonstrated the validity of the technique with an example visual search experiment where participants searched for “L”s among “T”s and then reported at the end of the trial whether 0, 1, 2, or 3 targets were present. The time-stamps of fixations on targets and distractors during search were used to extract short segments of pupil data, which were then baseline-subtracted, temporally aligned, and averaged together across all subjects. Data visualizations showed that target fixation elicited small transient pupil dilations, whereas distractor fixation did not. Refixation on targets had a similar effect, with the addition that dilation seemed to start around 1 second prior to the fixation. These findings were based on the average waveforms from 770 target fixations and 1511 off-target fixations across 17 subjects. No statistical analyses were reported and there was no indication of variability at the level of individual subjects and epochs, leaving the reliability and practicability of this technique open to question. Acknowledging the PFE in a separate communication, Klingner (2010b) argued that it may not have been problematic due to his use of a Tobii 1750 remote eye tracker, which measured pupil size as the major axis of an ellipse fitted to the image of the pupil. As long as changes in eye-to-camera distance were adequately accounted for by this system (e.g. scaled based on changes in interpupillary distance), pupil measures obtained on this principle should not be affected by optical distortion (although noncircular pupils would presumably still result in some small error).

Beyond the work of Porter et al. (2007) and Klingner (2010a) only a handful of other studies speak to the question of pupil dynamics in visual search. Privitera et al. (2010) and Wierda et al. (2012) echoed the effect of transient pupil dilation upon visual target detection observed by Klingner (2010a) but in both cases the task involved rapid serial visual presentation of stimuli without eye movements, which does not qualify as visual search in the strictest sense. Some further work was carried out by Porter and colleagues (Porter, Leonards, et al., 2010; Porter, Tales, et al., 2010) wherein pupillometry was used expressly as a measure of processing load to explore free-viewing search performance in aging and clinical populations, but in both cases, there was little focus on methodological confounds other than stimulus characteristics and limited discussion of what the pupil data may have represented in terms of task components and underlying brain activity. Finally, a study by Meghanathan, van Leeuwen, & Nikolaev (2015) involving search for multiple targets reported that fixation duration surpasses pupil size as an index of memory load, but here the pupil samples were taken primarily from within fixations and there appeared to be some confusion over how the system they used measured pupil size (the choice of pupil tracking mode with an EyeLink 1000 system does not affect the dependence of pupil size on gaze direction). Questions, therefore, remain: What causes the pupil to dilate during free-viewing visual search? Can meaningful data be acquired despite the significant methodological obstacles of stimulus confounds and the PFE? How reliable and practicable are these data? Moreover, how does it all relate to task demands and what is happening in the brain?

Inspired by the good work already done, this project aimed to make further ground on the general questions posed above. We devised a visual search task combining elements of the tasks used by Porter et al. (2007) and Klingner (2010a). Participants searched for target “Cs” among heterogeneously rotated distractor “Cs” and indicated the number of targets—0, 1, 2, or 3—via mouse click at the end of the trial. We allowed up to 10 seconds per search but also gave participants the option to terminate search via key-press if they purported to have found all of the targets. We predicted that this option would be used most frequently in trials with three targets, because the discovery of a third target in under 10 seconds might be taken as a cue to end the search and report the result, whereas accuracy in the other conditions would depend on a more thorough inspection of the items in the display. The optional button press was included so we could gauge the extent to which response preparation and execution contributed to pupil dilation around search offset. To explore pupil dynamics during search we used both “whole-trial” analysis (e.g. Porter et al., 2007) and fixation-aligned pupillary response averaging (Klingner, 2010a). The former was used to get a picture of the general pattern of pupil dynamics across the phase of search, and the latter to examine pupil modulations surrounding target and distractor fixations during search. We aimed to replicate and further evaluate Klingner's principal finding of pupil modulation following initial fixations and refixations on targets, and we also sought to assess the effect of successive target discovery in trials with multiple targets. We reasoned that if pupil dilation reflects memory load during search then, on average, it should be lowest for trials without targets and greatest for trials with three targets. Further, it should increase with successive target discovery, because efficient search depends on keeping track of previously located targets. Finally, to gauge the extent of the PFE and correct the visual search data for eye movement artifacts we implemented an adapted version of the ellipse tracking task described by Brisson et al. (2013).

## Method

Twenty-seven participants (19 women; age range = 18–31 years; *M* = 21.74; *SD* = 3.51) completed the experiment voluntarily or in exchange for course credit. All participants were students at Swansea University reporting normal or corrected-to-normal acuity and color vision. The experimental protocol was approved by the Ministry of Defence Research Ethics Committee and the Department of Psychology Ethics Committee at Swansea University. Written informed consent was obtained from each participant.

Participants completed the main visual search task in two sessions, both of which lasted approximately 1 hour and took place less than a week apart. Prior to each session, a short ellipse tracking task was administered to obtain data for measuring and correcting the PFE. Participants then went straight on to perform the visual search task.

### Ellipse tracking

**Figure 1.**
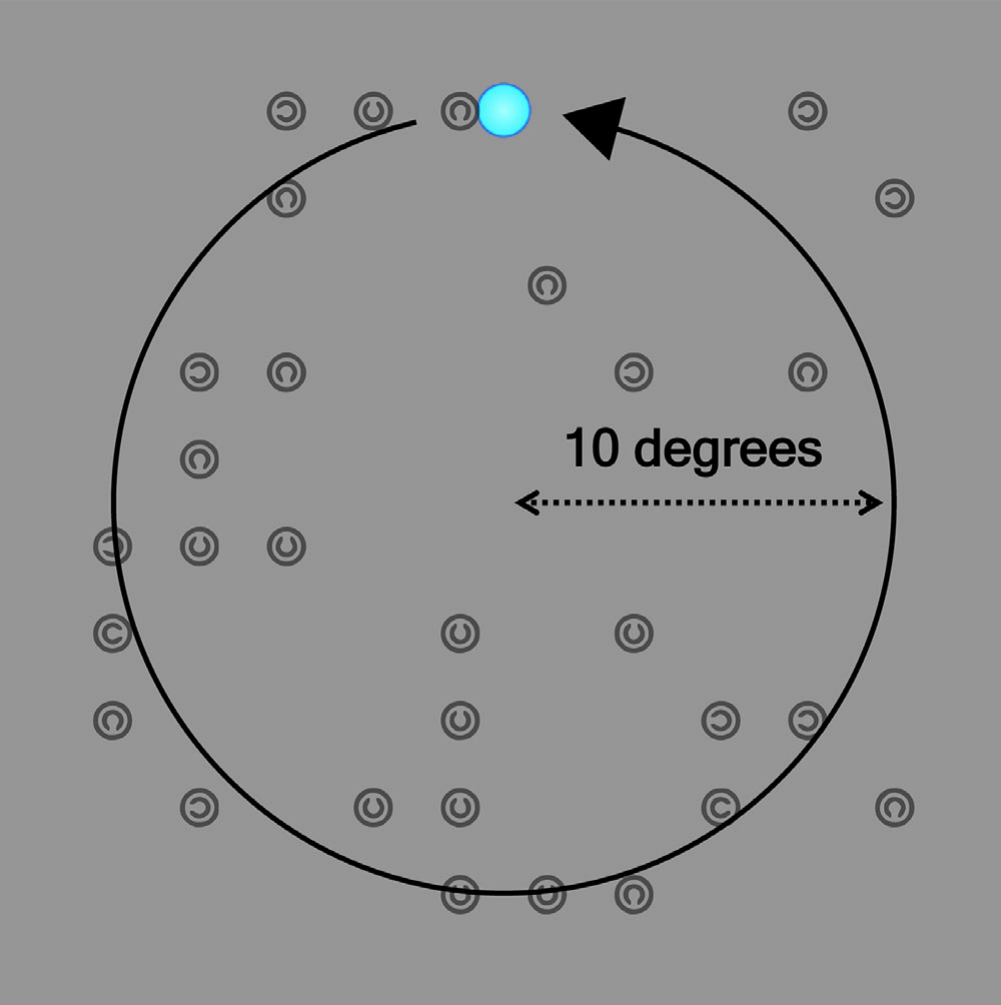
The tracking object in the ellipse tracking task, with its trajectory shown by a circular arrow (not to scale). The path of the object mapped the horizontal and vertical limits of the portion of the screen where display items (overlaid here for illustrative purposes) could appear in the visual search task.

The ellipse tracking task was a close replication of Brisson et al. (2013). Participants tracked a small blue ellipse (1.8° × 1.8°) for 30 seconds as it traced a circular pattern on a grey background counterclockwise around the screen (6 full rotations). The task is simply a convenient method for measuring pupil size across a range of *x* and *y* gaze coordinates on the screen. Crucially, the motion path of the ellipse mapped the horizontal and vertical limits of the portion of the screen where stimuli could appear in the visual search task (20° × 20°, Figure 1). Prior to the task, a 5-point calibration routine was performed. Participants then received on-screen instructions to fixate the ellipse in its starting position at the top of the screen and to track it as closely and accurately as possible without trying to anticipate its motion. Trials were initiated by the participant with a button press when they were ready to begin. Pupil area and gaze coordinates were recorded throughout. There were no changes in the geometry (e.g. eye level, eye-to-camera distance, eye-to-screen distance, etc.) of the experimental setup between the ellipse tracking and visual search tasks during each session.

### Visual search

The visual search task was a hybridized version of the tasks implemented by Porter et al. (2007) and Klingner (2010a). Participants searched for forward-facing “Cs” in arrays of heterogeneously oriented distractor “Cs” rotated through 90°, 180°, or 270°. A repeated-measures design was used, with the number of targets present in the search array as the only systematic experimental manipulation (Targets: 0, 1, 2, and 3). Set size was fixed at 30 display items for all trials, targets and distractors included. Targets and distractors subtended approximately 1 degree of visual angle and were placed randomly at predefined locations on a 10 × 10 virtual grid with a uniform grey background subtending approximately 20 × 20 degrees of visual angle at the viewing distance of 40 cm. The four locations at the center of the grid were excluded to avoid rapid identification of search elements appearing close to fixation at the beginning of a trial. Two targets could not appear in the same quadrant of the screen, and where distractor variants could not be present in equal measure (i.e. for arrays with 1 or 2 targets) they were chosen at random.

**Figure 2.**
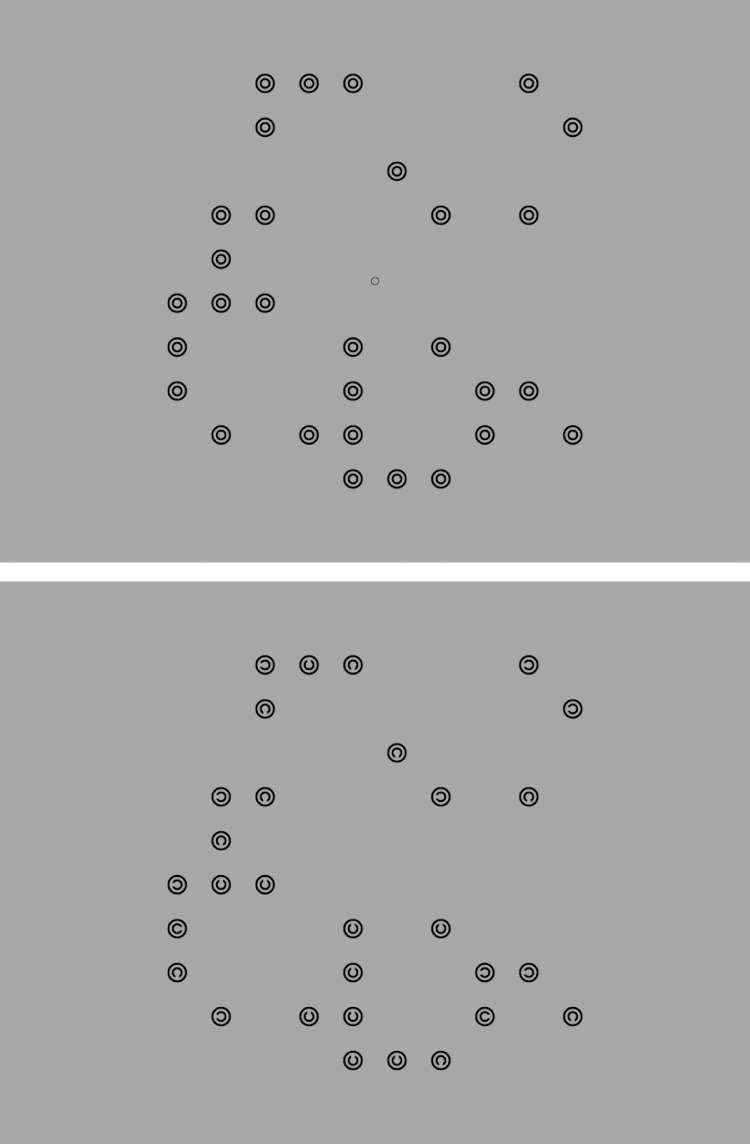
An example two-target search array (top) and its accompanying mask (bottom).

Following a 2 second fixation period and a 4 second mask, the arrays were displayed for 10 seconds or until participants opted to terminate the search by pressing the space bar with their subdominant hand, on belief of finding all targets. After another 2 second mask, participants indicated via mouse-click with their dominant hand the number of targets thought to be present in the array. The experiment consisted of 144 experimental trials spread evenly across two sessions completed less than a week apart. A 13-point calibration and validation routine for the eye tracker was performed at the beginning of the task and again as required throughout. At the beginning of each session, participants were instructed to perform the task as quickly as possible without compromising accuracy. Then they completed 4 practice trials (one for each level of targets, randomized) before undertaking 4 blocks of 18 experimental trials. The same set of 152 search arrays was used for each participant in the experiment (37 for each level of Targets), all of which were prepared in advance along with a luminance mask to match. Accuracy and search time (from search onset until termination or the maximum time of 10 seconds) were recorded for each trial, as well as continuous recordings of gaze position and pupil size. A drift check was performed before each trial to verify that the calibration model had not become grossly invalidated. Figures 2^3^ to 4 show an example search array for a two-target trial together with its mask, the general trial procedure, and example trial data with image and interest area overlay.

**Figure 3.**
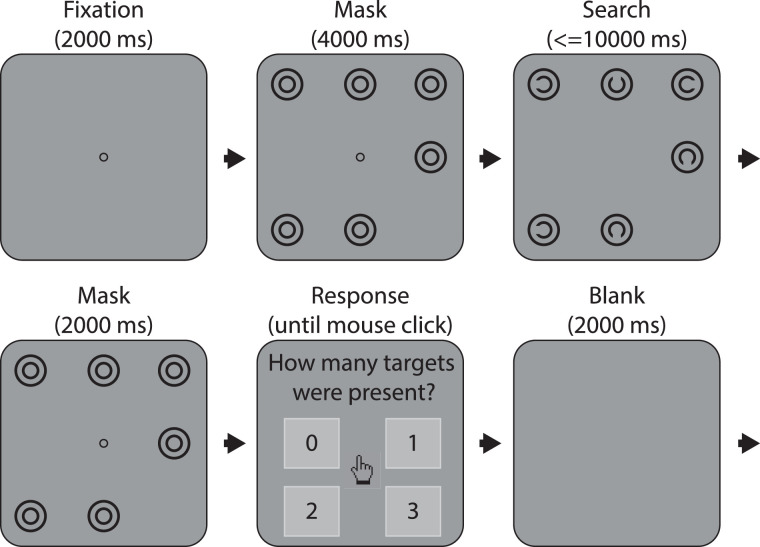
Schematic illustration of the trial sequence for the visual search task. Each trial began with a drift check followed by a 2000 ms fixation, then a mask. After 4000 ms, the mask was replaced by the search array, which remained on screen for either 10 seconds or until participants indicated they had finished the search by pressing the space bar. The mask then reappeared for 2000 ms prior to the response screen, where participants responded to the question “How many targets were present?” The trial cycle ended with a 2000 ms blank screen. Displays have been simplified for purposes of illustration.

**Figure 4.**
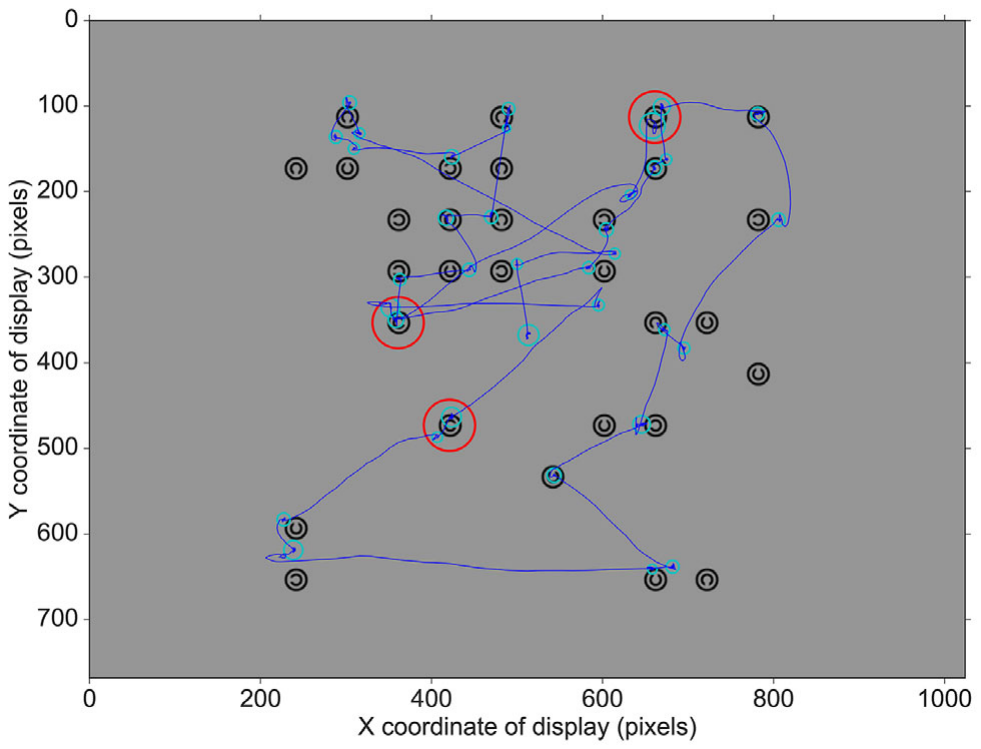
Example trial data for a three-target trial with image and interest area overlay. Gaze data are shown in dark blue, fixation data in light blue, with the size of the circles representing fixation duration. The red lines delimit the extent of the interest areas that were used to classify fixations as belonging to a particular item (shown here only for targets).

### Software and apparatus

All stimuli were presented on a 24-in. Ilyama monitor running at a resolution of 1024 × 768 (1:1 aspect ratio) with a refresh rate of 144 Hz. The screen and eye tracker were enclosed such that the only direct illumination came from the screen. A viewing distance of 40 cm was maintained by a chin rest and forehead bar. A colorimeter (ColorCAL MKII, Cambridge Research Systems) was used to measure the surface luminance of the grey background (73.56 cd/m^2^) and the surrounding dark light of the unused portion of screen (0.54 cd/m^2^). Pupil size and gaze data were recorded monocularly (left eye) using an EyeLink 1000 (SR Research, Mississauga, ON, Canada) system in tower mount configuration (35 mm lens) sampling at 1000 Hz. According to the user manual, pupil size measurements obtained with this system are resolved to within 0.2% of diameter and affected by up to 10% by pupil position (SR Research Ltd., 2010). Both tasks were programmed with Experiment Builder (SR Research).

### Data processing and statistical analysis

#### Preprocessing and analysis of pupil data

Pupil data were processed and analyzed using custom python scripts and *Cili* (Acland & Braver, 2014), an open-source eye tracking data tools package. Eye-blink periods were detected using Eyelink's standard parsing algorithm and reconstructed with linear interpolation. Often there were some samples after a blink that were still noticeably part of the blink artifact so we extended all blink end points to the first sample (up to a maximum of 1000 ms following the original end point) where the z-scored rate of change of the pupil time course dropped below 10% of the average within a 100 ms moving window. The average percentage of interpolated data during search was 5.15% (*SD* = 3.52%, minimum = 0.44%, maximum = 17.22%). In the 3 seconds prior to search onset and the 3 second post offset, it was 5.05% (*SD* = 6%, minimum = 0%, maximum = 31.46%) and 16.5% (*SD* = 16.34, minimum = 0%, maximum = 79.03%), respectively. The average proportion of samples interpolated for trials included in the analysis is plotted as a function of time alongside the pupil data for the “whole-trial“ analysis (i.e. in Figure 8.) No participants were excluded on the basis of blink rate or general quality of eye tracking data. After interpolation the data were downsampled to 50 Hz and, in line with a common standard (e.g. de Gee, Knapen, & Donner, 2014; Hoeks & Levelt, 1993; Hupé et al., 2009; Kloosterman et al., 2015), smoothed using a third order Butterworth filter (cut-off = 4 Hz) to remove background noise that does not convey cognitively meaningful information.

#### PFE analysis and correction

As per Brisson et al. (2013), the relationship between pupil size and point of gaze during the ellipse tracking task was quantified using multiple linear regression. This was done for each individual recording session using the formula:

(1) $$P^{'}=b_{0}+b_{1}X+b_{2}Y+e$$

Where *P'* is the estimated pupil size, *X* and *Y* are the horizontal and vertical gaze coordinates, and *e* is the residual error. The coefficients from the regression models for each session were then used to correct pupil size measurements from the ellipse tracking and visual search tasks as follows:

(2) $$P_{c}=P-b_{1}X-b_{2}Y$$

Where *P_c_* is the corrected pupil size, *P* is the recorded pupil size, *X* and *Y* are the relevant gaze coordinates, and *b_1_* and *b_2_* are the coefficients from the regression model.

#### Whole-trial pupil analysis

Whole-trial pupil analysis was conducted to assess the general pattern of pupil dilation and the effect of targets on pupil size during search. Average pupil traces for each level of targets were time-locked to search onset and offset. The data between these points were then normalized to account for differences in search time, a method that aims to ensure that pupil traces are compared at roughly equivalent points of processing (Porter et al., 2007). The non-self-terminated trials, which share a common time-base during search, were additionally analyzed separately. Pupil data were expressed as %-change from baseline, with the baseline calculated as the mean pupil size across 1000 ms prior to search onset. Only trials that resulted in a correct response were included in the analysis.

#### Fixation-aligned pupillary response averaging

The average pupil traces across the phase of performance may give insight into the typical fluctuations in processing load during search but they cannot show how the pupil responds to individual task components which occur at unpredictable times within a trial, such as fixations on targets and distractors, and refixations on previously visited items. To gain insight into the processing that accompanies these subtasks and how it may differ between subjects and throughout the course of a search, we used fixation-aligned pupillary response averaging (Klingner, 2010a), where subtask epochs are identified from gaze data, temporally aligned, and then averaged together. We were initially interested in replicating Klingner's finding of transient pupil dilation following target fixation and refixation, and statistically quantifying the effects of pupil modulation. We then aimed to gain insight into the consistency of these findings at the level of individual epochs and individual subjects. To these ends we used the timestamps from fixations on targets and distractors to index and align short (-500:2000 ms) segments of pupil data. The data were expressed as %-change from the average of a 500 ms baseline prior to fixation onset.

Fixations were identified using EyeLink's standard event parsing algorithm. Accordingly, any sample that did not exceed the motion (0.1°), velocity (30°/sec) or acceleration (8000°/sec^2^) thresholds for saccade detection was deemed to be part of a fixation. After data collection, brief fixations (< 100 ms) were merged with neighboring fixations (< 1° visual angle), or discarded. The remaining fixations were then assigned to display items if they fell within 1.25°. We extracted epochs only for fixations whose duration was greater than or equal to 120 ms and we additionally discarded all fixations whose duration was above 3 SDs from the mean of all target fixations. Finally, fixations which occurred within 1000 ms of search onset and 3000 ms of a button-press used to terminate search were discarded to avoid contamination of the pupil waveforms by display changes and participant interaction. We only analyzed data from trials that resulted in a correct behavioral response.

First, we sought to replicate Klingner's effect of initial target discovery. To do this we extracted fixation-aligned pupillary responses for all valid target fixations in trials with one to three targets and calculated the grand averages. These were compared with the averages for valid distractor fixations (selected from trials without targets). Second, we looked at the effect of refixation on targets and distractors. Here, we used the same selection criteria as previously but with the added condition that refixations occurred at least 1000 ms after the initial fixation. Finally, we looked at the effect on pupil size of successive new-target fixation in trials with three targets.

It is noteworthy that an implicit assumption of this method is that the object of fixation is recognized, or at least processed sufficiently as to enable the participant to perform the task correctly. This could not be controlled for rigorously, but the minimum fixation duration threshold of 120 ms and the analysis of fixation-aligned responses only from trials with correct behavioral outcomes will have helped to maximize the likelihood that the assumption held true for each fixation.

#### Statistical analysis

Performance measures and pupil averages were analyzed using separate one-factor ANOVAs (*a* = 0.05). Where appropriate, *p* values were adjusted for violations of sphericity using the Greenhouse-Geisser correction. The statistical evaluation of pupil modulation from baseline and differences in modulation between conditions for each subject's average pupil traces were assessed using two-tailed nonparametric permutation tests with cluster-based correction for the multiple comparisons problem (Maris & Oostenveld, 2007). This approach has the advantages of sensitivity without depending on theoretical assumptions about the data, and it reduces experimenter bias associated with choosing an epoch over which to calculate summary statistics. *t*-tests were used for comparisons between two conditions (e.g. target versus distractor) with the significance thresholds for test statistics determined theoretically from the appropriate degrees of freedom at *a* = 0.05.

## Results

### Ellipse tracking task

**Figure 5.**
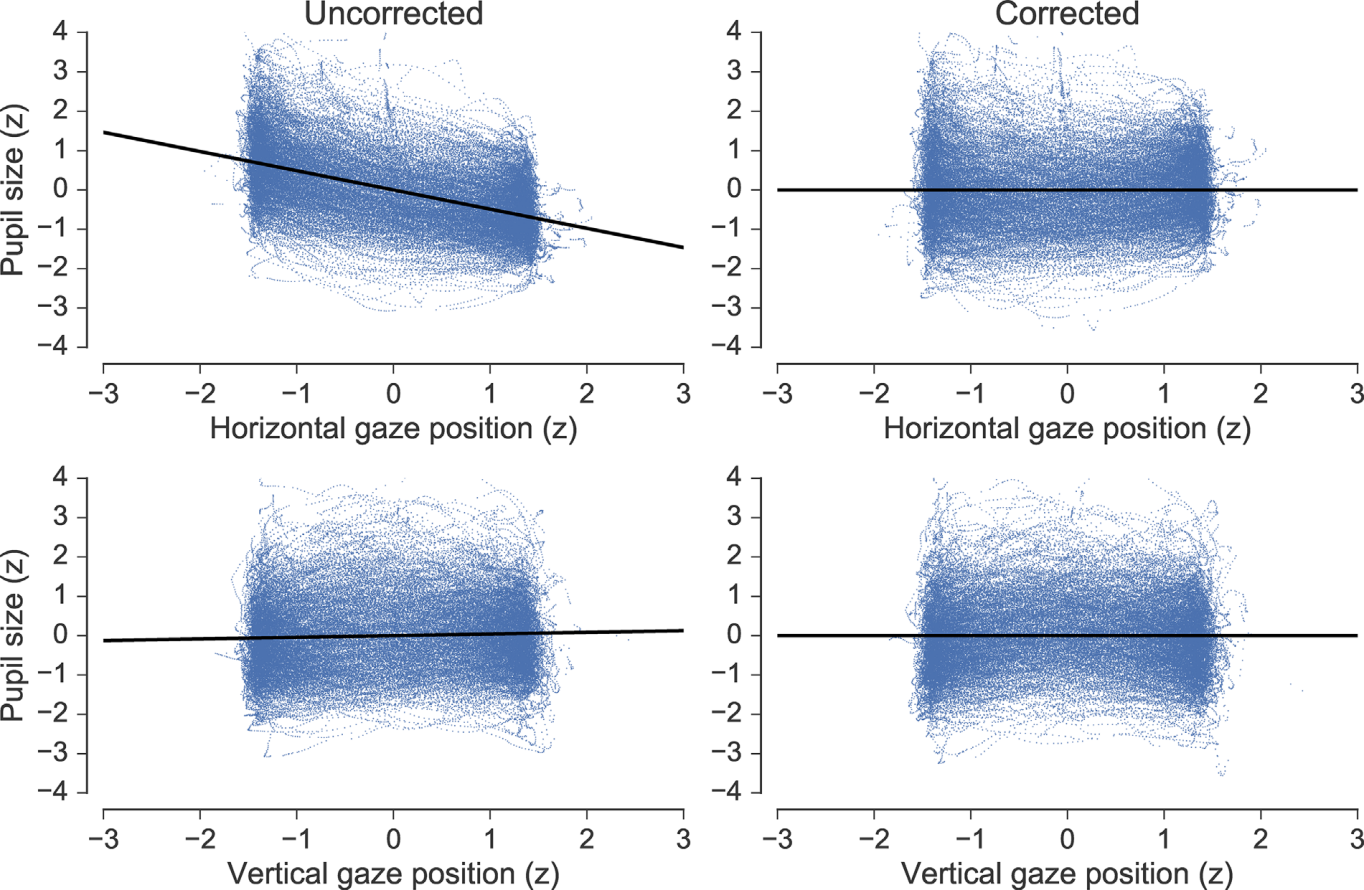
Pupil size across all sessions in the ellipse tracking task as function of horizontal (top row) and vertical (bottom row) gaze position. Uncorrected pupil data are shown in the left column and corrected data in the right column.

**Figure 6.**
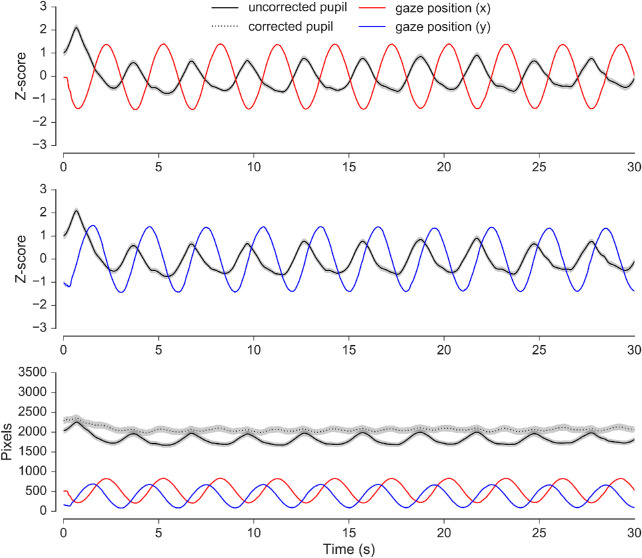
Pupil size and gaze position as a function of time in the ellipse tracking task (averages across sessions). The top and middle panels illustrate the covariance between pupil size and horizontal and vertical gaze position, respectively. The bottom panel shows pre- and post-corrected pupil data together with the horizontal and vertical gaze coordinates in pixel units. The covariance between pupil size and gaze position is noticeably diminished after the correction was applied. Shaded areas surrounding the pupil traces reflect the SEM (bootstrapped, 5000 iterations).

A significant regression equation was found for all sessions, with all predictors being significant (all p values < 0.05). Individual regression models are not explored in detail as they simply reflect factors that were not under experimental control, such as between-session differences in geometrical layout, idiosyncratic pupil shapes, and the average participant pupil size in native pixel units for each individual recording session. The average parameters across all regression models, however, can give some general insight into the pattern of the PFE. On average, 34.36% of variance (mean *R2* = 0.343, ranging from 0.048 to 0.734, *SD* = 0.182) in pupil data was accounted for by changes in *X* and *Y* gaze coordinates. The average regression coefficient for *X* was -0.483 (ranging from -1.146 to -0.122, *SD* = 0.276), and for *Y* it was 0.009 (ranging from -0.712 to 0.518, *SD* = 0.257). From this it can be gleaned that pupil size was affected mostly by changes in horizontal gaze position and that rightward horizontal shifts in gaze were always associated with decreasing pupil size. This is appropriate given that pupil size was always recorded from the left eye, making the pupil position consistent between sessions with respect to the optical axis of the camera. On the other hand, changes in vertical gaze position caused the pupil to either increase or decrease in size, depending on the session. This can be linked to differences in eye level arising from session-specific adjustments to the chin rest of the tower-mount, which were practically necessary to enable eye tracking and ensure participant comfort. The findings described above are illustrated clearly in Figures 5 and 6—the former shows corrected and uncorrected pupil size data for all sessions (with regression lines) as a function of horizontal and vertical gaze position and the latter shows grand averages of pupil size and gaze position between all sessions as a function of time. The effect of applying the correction with Equation 2 to the ellipse tracking data is visualized in the right-hand panels of Figure 5 and the bottom panel of Figure 6.

### Visual search task

#### Behavioral data

**Figure 7.**
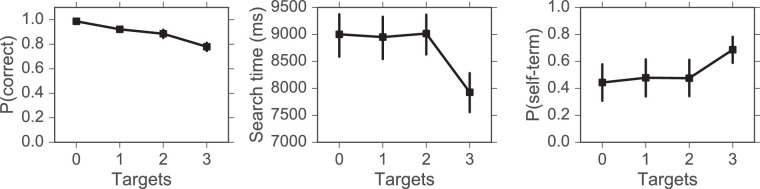
Performance data for visual search: Search time (left), accuracy (middle) and the proportion of self-terminated trials (right) as a function of Targets. Error bars represent 95% confidence intervals (bootstrapped, 5000 iterations).

One participant was excluded from all subsequent analyses for terminating 100% of trials and attaining only chance accuracy. All remaining participants attained at least 78% accuracy overall (*M =* 89.17%, *SD* = 4.88%) and at least 55% (*SD =* 10.25%) accuracy across each level of targets. Our task differed from canonical laboratory-based visual search tasks in that multiple targets could be present and participants could terminate a search via key-press when they were ready to report the number of targets (otherwise the search would time-out after 10 seconds). The performance measures were, therefore, search-time, accuracy, and the proportion of self-terminated trials. The averages for these measures across all 26 participants included in the analysis are presented in Figure 7.

Separate one-way ANOVAs and Bonferroni-corrected follow-up tests (Table 1) were conducted for each of the performance measures. Targets had a significant main effect on accuracy, *F*(2.33, 58.13) = 56.94, *p* < 0.001, *η_p_^2^* = 0.7 (see Figure 7, left), which was characterized by a linear downward trend ranging from close to 100% for trials with 0 targets down to approximately 80% for trials with 3 targets. Accuracy did not differ significantly between trials with 1 or 2 Targets (*p* = 0.17), but all other pairwise comparisons were significant (all p values < 0.008).

**Table 1.** Follow-up test results for performance measures.

Targets	*MD*	*CI*	*t*	*p*	*d*
Accuracy					
0 vs. 1	0.065	0.021, 0.109	4.01	<0.008	0.79
0 vs. 2	0.101	0.057, 0.146	6.25	<0.008	1.23
0 vs. 3	0.207	0.163, 0.251	12.76	<0.008	2.50
1 vs. 2	0.036	−0.008, 0.08	2.24	0.170	0.44
1 vs. 3	0.142	0.098, 0.186	8.75	<0.008	1.72
2 vs. 3	0.106	0.062, 0.15	6.51	<0.008	1.28
Search time					
0 vs. 1	50	−258, 360	0.45	0.357	0.09
0 vs. 2	−7	−317, 302	−0.07	0.929	−0.01
0 vs. 3	1377	1067, 1687	12.05	<0.008	2.36
1 vs. 2	−58	−368, 251	−0.51	0.329	−0.10
1 vs. 3	1326	1016, 1636	11.61	<0.008	2.28
2 vs. 3	1385	1075, 1694	12.12	<0.008	2.38
P(self-term)					
0 vs. 1	−0.035	−0.152, 0.081	−0.82	0.118	−0.16
0 vs. 2	−0.038	−0.154, 0.079	−0.87	0.274	−0.17
0 vs. 3	−0.320	−0.436, −0.203	−7.44	<0.008	−1.45
1 vs. 2	0.002	−0.119, 0.114	−0.05	0.920	−0.01
1 vs. 3	−0.284	−0.401, −0.168	−6.62	<0.008	−1.29
2 vs. 3	−0.282	−0.399, −0.166	−6.57	<0.008	−1.28

CI = 95% confidence interval for mean difference, d = Cohen's d; MD = mean difference.

Tests were conducted using a Bonferroni adjusted alpha level of 0.008 (0.05 / 6) for each family of six tests.

For search time, there was a significant main effect of Targets, *F*(1.58, 39.48) = 71.2, *p* < 0.001, *η_p_^2^* = 0.74 (see Figure 7, middle). Follow-up tests confirmed the source of this effect—search time for trials with three targets was significantly shorter than for trials with any other number of targets (all *p* values < 0.008). No further significant differences were found.

Finally, the main effect of targets on the proportion of self-terminated trials was significant, *F*(1.63, 40.66) = 23.94, *p* < 0.001, *η_p_^2^* = 0.49 (see Figure 7, right). For trials with 0, 1, or 2 targets, the proportion of self-terminations was near 50%. There were significantly more self-terminations for trials with three targets than for trials with any other number of targets (all *p* values < 0.008) but no other differences were significant. The large 95% confidence intervals (CIs) for this measure reflect the considerable differences in search strategy between subjects.

#### Pupil data

**Figure 8.**
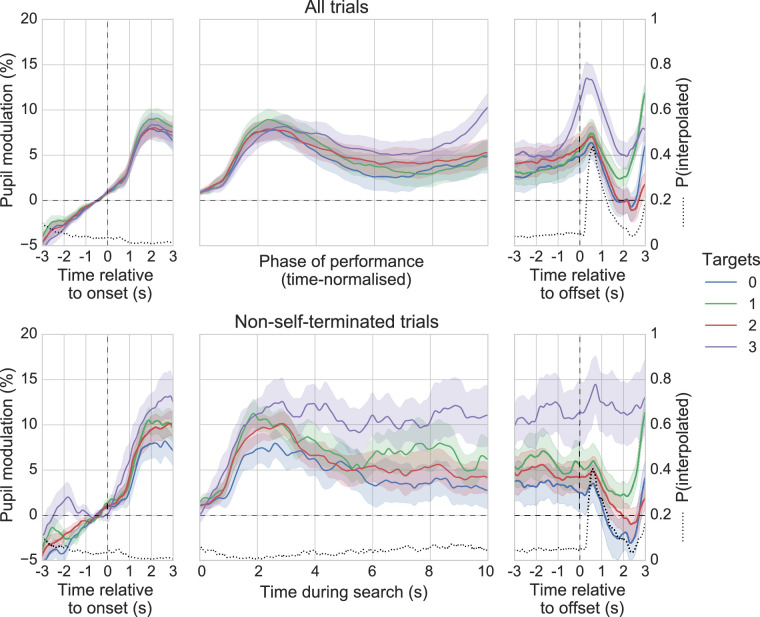
Top: Between-subject grand averages for each level of Targets, showing pupil size relative to onset (left), phase of performance (middle, time-normalized) and offset (right). Bottom: As previous but showing data only for trials not terminated via button press (i.e. trials with a search time of 10 seconds). Shaded areas surrounding each pupil trace reflect the SEM (bootstrapped, 5000 iterations). The proportion of interpolated pupil data (dotted black line) is additionally displayed for axes expressing time in seconds.

**Figure 9.**
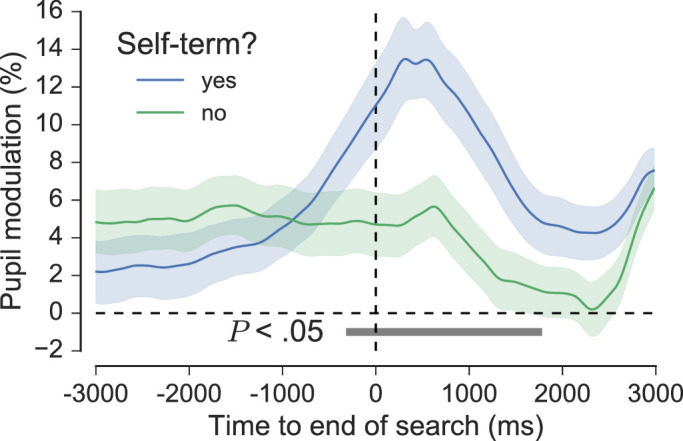
Average pupil traces for all participants at search offset, grouped by whether the trial ended with a button press or not. The horizontal grey bar indicates significant differences between the two traces, as revealed by nonparametric permutation tests (1024 permutations, *p* < 0.05, cluster-corrected for multiple comparisons). Shaded areas surrounding each pupil trace reflect the SEM (bootstrapped, 5000 iterations).

Whole-trial analysis of pupil data was first conducted to gain insight into the general pattern of pupil modulation across the phase of search performance. The top panel of Figure 8 shows the grand average pupil traces for each level of Targets, time-locked to search onset, search performance (time normalized), and search offset. Pupil size increased prior to onset and then continued to increase once the search began, peaking at around 2000 ms into the search. After this initial peak, the pupil began to decrease in size, but then it increased again toward the end of the search. All conditions followed this general pattern—similar to that observed by Porter et al. (2007) and Meghanathan et al. (2015)—although there was a more pronounced dilation and subsequent peak at search offset for three-target trials compared with all other trial types. This effect can be attributed to self-termination via button-press, which occurred more frequently in trials with three targets (right panel of Figure 8). To confirm this, we grouped the pupil traces at search offset by whether the trial was self-terminated or not. On trials that were self-terminated, the pupil began to dilate at least 1000 ms prior to search offset and peaked shortly afterward. This pattern differed significantly from trials that were not self-terminated, where pupil size remained relatively constant around search offset (*p* < 0.05, cluster-corrected permutation test, Figure 9). This finding can be attributed to the effects of the decision to terminate the search and the subsequent motor act, both of which have been observed to elicit pupil dilation in similar contexts (e.g. Einhäuser et al., 2010; Richer & Beatty, 1985). It should be noted that the pupil data following search offset are somewhat distorted due to an increased proportion of interpolated data (i.e. a high blink rate), and that this appears to account for the small transient peak in pupil size that is present for all conditions at around 600 ms post-offset.

A one-factor ANOVA conducted on the average pupil modulation across the phase of performance (time-normalized) revealed no significant main effect of targets (*p* > 0.05). To gain further insight into the pattern of pupil dilation across the whole of search we replicated the previous visualization and analysis using only non-self-terminated trials, which share the same time base during search (see Figure 8, lower panels). Two further participants were excluded from this analysis due to their having some empty cells in the design. Although similar in their overall shape to the data presented for all trials, these data appear noisier and the traces do not slope upward as they approach search offset. A one-factor ANOVA conducted on the average pupil modulation during search showed a significant main effect of targets, *F*(1.61, 37.17) = 4.36, *p* < 0.01, *η_p_^2^* = 0.16. Bonferroni-corrected follow-up tests confirmed that average pupil size for trials with 3 targets (*M* = 10.43, *SD* = 10.37) was significantly greater than for trials without targets (*M* = 4.6, *SD* = 7.26), *t*(23) = 3.36, *p* < 0.008, 95% CI = -10.62 to -1.12, Cohen's *d* = 0.69). No other tests were significant.

**Figure 10.**
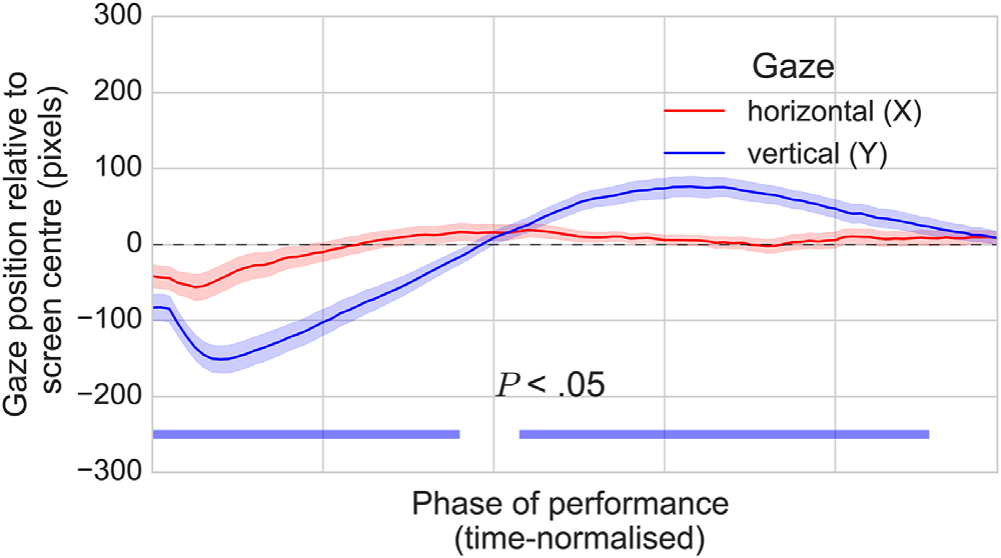
Average horizontal and vertical gaze position across the phase of visual search performance (time-normalized). The pattern of vertical gaze position suggests that participants generally favored a left-to-right / top-to-bottom strategy for viewing the stimuli. Shaded areas surrounding each pupil trace reflect the SEM (bootstrapped, 5000 iterations).

Although pupil data were corrected for gaze position using the regression model from the ellipse tracking routine specific to each session, they may have been affected by error that was not captured by the model and which may have been rendered systematic by regularities in participant viewing behavior. To check whether participants consistently viewed the search arrays in a particular way, the average horizontal and vertical gaze coordinates across the phase of performance were calculated. If viewing behavior was mostly random, then horizontal and vertical gaze coordinates would not deviate significantly from the center of the screen. Figure 10 clearly shows this not to be the case. From the pattern of gaze position it can be inferred that participants generally favored a left-to-right / top-to-bottom search strategy, meaning that any variability in pupil size arising from changes in horizontal or vertical gaze position that was not accounted for by the correction procedure may have systematically affected the observed pupil responses.

**Figure 11.**
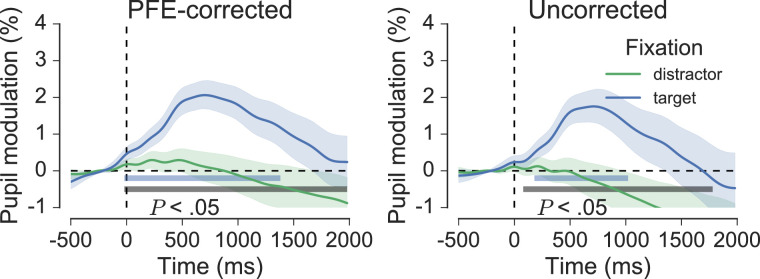
Grand-average fixation-aligned pupillary responses for target and distractor fixations, shown for PFE-corrected (left) and uncorrected pupil data (right). The horizontal blue bars denote significant modulation from baseline, and the grey bars indicate significant differences between the colored traces, as revealed by nonparametric permutation tests (1024 permutations, *p* < 0.05, cluster-corrected for multiple comparisons). Shaded areas surrounding each trace reflect the SEM (bootstrapped, 5000 iterations). Significance was not dependent on the data-driven PFE correction procedure.

To gain a greater level of insight into the cognitive pupil dynamics during search, fixation-aligned pupillary response averages were calculated for all target and distractor fixations, which fit the selection criteria described previously. The across-trial effect of target fixation reported by Klingner (2010a) was, here, replicated at the group-level, between participants (Figure 11). Target fixations lead to significant pupil modulation, which, on average, reached peak of 2.02% at a latency of 680 ms from fixation onset, returning to baseline levels after around 2000 ms. This effect differed significantly from the effect of distractor fixation, which was characterized by relatively stable pupil responses and no significant change from baseline (*p* < 0.05, cluster-corrected permutation test, Figure 11). It is noteworthy that the effect of target fixation was present even in data that were not corrected for pupil foreshortening (see right panel of Figure 11).

**Figure 12.**
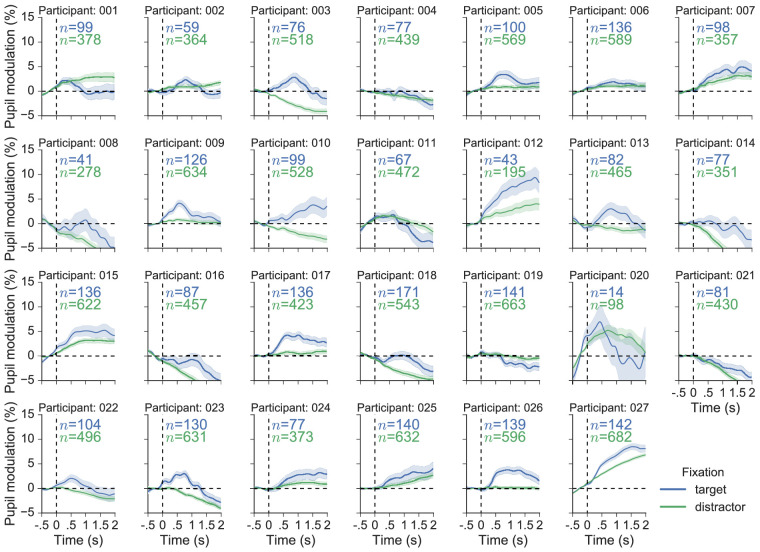
Variability at participant level for fixation-aligned pupil responses associated with fixations on targets and distractors. The colored numbers on each axis denote the number of epochs that were used to compute the average waveform (which is the number of fixations that remained after the selection process). Shaded areas around the colored traces show the SEM (bootstrapped, 5000 iterations). The data for subject 20 is displayed, although it was not included in the analysis.

**Figure 13.**
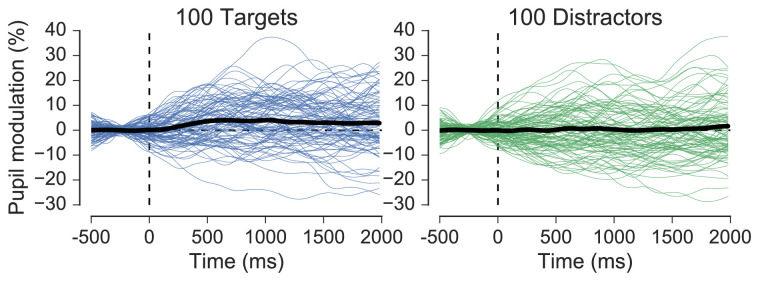
Single-trial variability for target (left) and distractor (right) fixations in one subject with prototypical averages (participant 17). One hundred target and distractor epochs were chosen at random and visualized, with their respective averages shown in black.

To get a further idea of the reliability of the effect of target versus distractor fixation it was visualized separately for each participant (Figure 12). Twenty-two of the 26 participants included in the analysis showed pupil responses that were greater on average for target fixations compared to distractor fixations, and at least 11 of these displayed the characteristic peak that was present in the grand-average waveform for target fixations. Finally, single-trial reliability was examined by selecting one participant who displayed prototypical pupil responses to targets and distractors (participant 17) and visualizing 100 randomly selected target and distractor epochs (Figure 13). This perspective on the data shows the considerable variability in the individual epochs that contributed to the participant averages, much of which is likely to have arisen from gaze position artifacts unaccounted for by the PFE-correction procedure.

**Figure 14.**
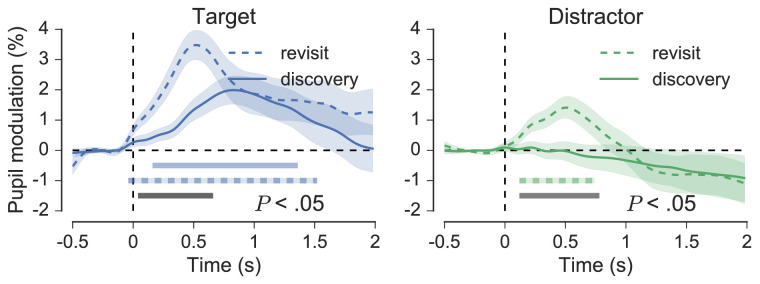
Grand-average pupil responses for target (left) and distractor (right) discoveries and revisits. Shaded areas surrounding each trace reflect the SEM (bootstrapped, 5000 iterations). Horizontal colored bars indicate clusters of significant modulation from baseline and grey bars indicate significant differences between the colored traces, as revealed by nonparametric permutation tests (1024 permutations, *p* < 0.05, cluster-corrected for multiple comparisons).

After comparing the effect of target and distractor fixation, fixations were split into discoveries (initial fixations on a target) and revisits (refixations on a target). Klingner (2010a) observed pupil dilation almost 500 ms prior to fixation onset for target revisits. This effect is not replicated here, but the responses for target discoveries and target revisits were significantly different (*p* < 0.05, cluster-corrected permutation test, left panel of Figure 14). On average, pupil responses to target revisits reached peak modulation of 3.41% at a latency of 520 ms from fixation onset, whereas target discoveries reached peak modulation of 2.01% at a latency of 840 ms. Due to noise in the data, differences in peak modulation and latency were not suitable for between-subject analysis. Interestingly, distractor revisits also caused significant modulation, which, on average, reached peak of 1.47% at a latency of 500 ms from fixation onset (*p* < 0.05, cluster-corrected permutation test, right panel of Figure 14).

**Figure 15.**
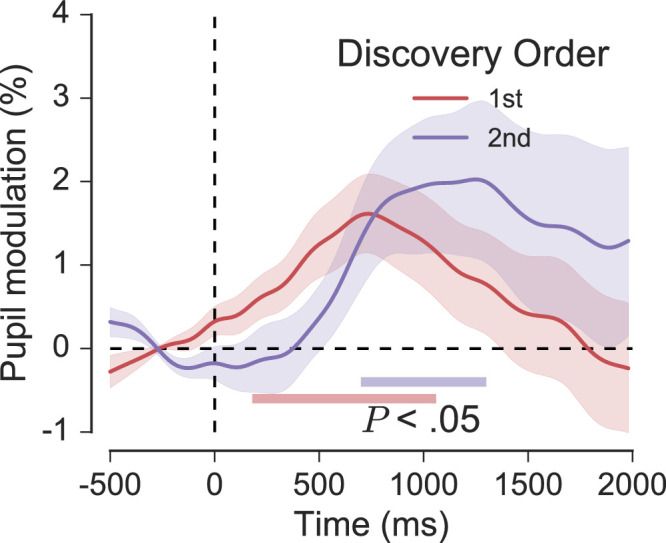
Fixation-aligned pupil responses for first and second target discoveries in trials with multiple targets. Shaded areas surrounding each trace reflect the SEM (bootstrapped, 5000 iterations). Horizontal colored bars indicate clusters of significant modulation from baseline, as revealed by nonparametric permutation tests (1024 permutations, *p* < 0.05, cluster-corrected for multiple comparisons).

Finally, to see if fixation-aligned responses were sensitive to the succession of target discovery during search, target fixations from trials with multiple targets were grouped by the order in which they were discovered (Discovery Order: first and second). Only first and second target discoveries were analyzed because third target discoveries were most often followed closely by a button response. The effect of Discovery Order is shown in Figure 15. Pupil responses associated with first and second discoveries differed most consistently between 0 and 500 ms after fixation, but the *p* value associated with this difference was not significant (*p* > 0.05).

## Discussion

This study sought detailed insight into the cognitive pupil dynamics of free-viewing visual search. Participants performed multiple target searches and reported the number of targets (0, 1, 2, or 3) via mouse-click at the end of each search, which lasted for 10 seconds or until it was optionally terminated with a button press. Whole-trial analysis of the pupil data for all trials (with time-normalized phase of performance) revealed that all conditions followed the same general pattern of pupil size changes comparable to those observed in previous studies of a similar nature (Meghanathan et al., 2015; Porter, Leonards, et al., 2010; Porter, Tales, et al., 2010; Porter et al., 2007), the most notable features being an initial peak in the first half of the search, a gradual dilation in the second half of the search, and substantial dilation around search offset when trials concluded with a button-press. For trials that were not self-terminated (i.e. those lasting 10 seconds) the pupil data followed a similar pattern, although trials with three targets had greater dilation on average and there were no positive slopes at search offset. Additionally, fixation-aligned pupillary response averaging revealed small transient pupil dilations following initial fixations on targets but not distractors, corroborating the study by Klingner (2010a), and also similar findings from rapid-serial-visual-presentation studies (Privitera et al., 2010; Wierda et al., 2012). We now discuss what these findings could mean in relation to the demands of the task and what might be happening in the brain, but first we focus on the visual search performance data and the pupillometry methodology used to counter visual stimulus confounds and the PFE.

### Performance data

The performance measures for the visual search task are in keeping with findings from previous studies of multiple target search and with what can be expected given the demands of the task. Accuracy decreased as targets increased, which can be explained in terms of the well-characterized phenomenon of subsequent search misses (Adamo, Cain, & Mitroff, 2013; Biggs, 2017; Cain, Adamo, Mitroff, Cosson, & Dash, 2013; Gorbunova, 2017). According to Cain et al. (2013), subsequent search misses arise primarily from scanning errors (i.e. failure to fixate the target during the search period), but are also associated with the cognitive effects of targets that have already been found (e.g. depletion of working memory (WM) resources that could have been used for further search), and to a lesser extent, with recognition, decision and strategy errors. Also observed in the current experiment was a reduced search time and increased proportion of termination-searches for three-target trials compared with all other conditions. These findings can be understood in terms of the task demands and the general strategy adopted by participants. It was known in advance that up to three targets could be present on every trial, meaning that accurate responses depended on finding either three unique targets or on exhaustively inspecting each item in the search array. If three unique targets were found before all of the items were inspected, participants could immediately terminate the search and provide the correct answer with relative confidence. Without the discovery of a third target, accuracy always depended on a thorough inspection of the array, leading to longer search times and a reduced proportion of termination searches.

### Stimulus confounds and the PFE

We wanted to be sure that the pupillometric effects of interest reflected underlying cognitive processes involved in search and to rule out, as far as possible, the effects of visual stimulus confounds and the PFE. In terms of visual stimuli, the most problematic issue is luminance. To minimize the effect that this would have on the pupil we followed the example of Porter et al. (2007) and used low-contrast grey-scale displays with targets and distractors that differed only in terms of orientation and not some other feature that could affect pupil size. The visual input was effectively identical on each trial and the arrays were masked prior to each search to minimize the effect of the luminance decrement associated with their appearance. Overall, these precautions should have ensured that the pupil data during search were sufficiently free from luminance artifacts. The rising baselines prior to search (see left panel of Figure 8) can be attributed in part to the luminance decrement associated with the appearance of the stimulus mask on the uniform grey background, but the fixed 4000 ms duration of the mask may have afforded accurate anticipation of the appearance of the stimuli, something which is also likely to have contributed to the dilation (Kahneman & Beatty, 1966, 1967). A simple jitter routine might have eliminated this latter component.

To account for the PFE, we implemented an adaptation of the data-driven correction routine established by Brisson et al. (2013). As expected, despite the minimal cognitive demands of the ellipse-tracking task, pupil size was found to vary systematically as a function of gaze position throughout the 30 second recording, showing sinusoidal variation, which closely mirrored the sine and cosine functions of the path of the ellipse (see Figure 6). Regression analyses revealed that pupil size in every session was significantly predicted by the *X* and *Y* dimensions of gaze position. In our implementation, an average of 34.36% of the variance in pupil size was explained by changes in gaze position, which is considerably higher than the value of 9.9% reported by Brisson et al. (2013). The difference here can be linked to two factors. First, the trajectory of the tracking object in this implementation spanned twice as many degrees of vertical visual angle and 7° more horizontal visual angle, which ensured larger deviations in gaze position relative to the optical axis of the camera, and, therefore, larger PFE. Second, Brisson et al. (2013) measured pupil diameter using an EyeLink 1000 system in desktop configuration with a 25 mm lens, whereas in the current experiment pupil area was measured using the same system but in tower-mount configuration with a higher resolution 35 mm lens. This means that the average recorded pupil size in pixel units for this experiment was most likely larger than it was for Brisson et al.'s experiment, which would lead to higher covariance (Brisson et al. did not report pixel measurements so this cannot be verified).

After correcting the pupil data for the ellipse tracking task using Equation 2 its covariance with gaze position was noticeably diminished. The flattening of regression slopes in Figure 5 and the reduced amplitude of sinusoidal variation in corrected pupil size in the bottom panel of Figure 6 both illustrate this effect. These observations supported the use of the regression models for correcting the visual search pupil data for gaze position artifacts on a trial-by-trial basis within each session, as the experimental geometry did not change between tasks. The effect of applying this correction was, however, unremarkable, making only a small difference to the grand averages and no difference to the reported significance of any statistical outcomes (e.g. see Figure 11). This implies that, to the extent that it encouraged random eye movements, the random distribution of display elements in the search arrays may have been a sufficient countermeasure to the PFE per se. Of course, the gaze patterns were not completely random, and the clear left-to-right and top-to-bottom preference during search may well account for some of the overall shape of the pupil data across the phase of performance, as well as the noise observed at the level of single fixation epochs for the effect of target and distractor fixation (see Figure 13). However, overall, it seems that the PFE was random enough so as not to distort experimental effects revealed by the whole trial and fixation-aligned averaging procedures. Applying the correction simply had the effect of cleaning the data, improving the appearance of the grand averages.

From a more general standpoint, a limitation of this data-driven approach to correcting pupil data is its implicit assumption that pupil size does not actually depend on gaze position and that any observed relationship is consequently artifactual. As Mathôt, Fabius, Van Heusden, & Van der Stigchel (2018) point out, this assumption may be flawed. For example, if participants are required to make uncomfortable eye movements to foveate extreme parts of the screen, the pupil may dilate because of the mental and physical effort involved with this action. Observing such effects in a saccade and fixate task, Mathôt, Melmi, & Castet (2015) opted against data-driven correction, as in this case it would have corrected true cognitive effects as though they were artifactual. Another issue specific to the current implementation is that participants began trials with a button-press, the pupillometric effects of which are noticeable at the beginning of trials. As this may have compromised the quality of the regression analyses, a more optimal solution would be to omit the button press, or have the motion pattern begin following a variable delay after a button press.

In summary, we believe that our rigorous control over stimulus confounds and detailed analysis and correction of the PFE justifies speculation on the cognitive origins of the pupillometric effects revealed by the averaging techniques.

### Pupil dilation, behavioral context, and the brain

Pupillometry is traditionally used in experimental psychology as a measure of “cognitive load,” “processing load,” “processing effort,” or some similar physical analogy for describing mental states during problem solving (e.g. Beatty, 1982b; Einhäuser et al., 2010; Kahneman, 1973; Laeng et al., 2012). Such concepts can be useful but they are also vague, encompass a broad range of cognitive functions, and ultimately derive their practical definitions from the methodological and behavioral contexts in which they are measured. Previous pupillometric studies of visual search have typically worked with these analogies (Porter, Leonards, et al., 2010; Porter, Tales, et al., 2010; Porter et al., 2007), but some have gone further and used pupillometry expressly as a measure of memory load (Meghanathan et al., 2015) or suggested that memory processes are the best explanation of the pupillometric effects observed (Porter et al., 2007). There is of course good evidence to suggest that visual search involves memory (e.g. Gibson et al., 2000; Gilchrist & Harvey, 2000; Kristjánsson, 2000; McCarley et al., 2003; Petersen et al., 2001; Shen et al., 2014; Shore & Klein, 2000) but also that it involves other aspects of cognition, which may induce pupil dilation, such as visual detection (Privitera et al., 2010; Wierda et al., 2012) and perceptual, and selective decision processes (Einhäuser et al., 2010, 2008).

In our data, average pupil size during search was greatest overall for trials with three targets, and for non-self-terminated trials it was significantly greater when three targets were present as compared to when no targets were present. This suggests that more processing effort was required as the number of targets increased. If more targets meant more effort, this could well have been due to the extra load they placed on working memory, as remembering the locations of already-discovered targets was requisite for efficient task performance (e.g. to avoid missing or double counting a target). The main effect of Targets during search was, however, statistically significant only for the analysis with non-self-terminating trials, whose power was reduced by further participant and trial exclusions. Various noncognitive factors could also have influenced the shape of the pupil responses for each condition. For example, average search time for three target trials was significantly lower than it was for 0 to 2 target trials, which will have led to the pupil traces being warped more by the time-normalization process in this condition. Three target trials were also more frequently terminated with a button press, the dilatory effects of which are well documented in the pupillometry literature (Hupé et al., 2009; Kloosterman et al., 2015; Privitera et al., 2014, Privitera et al., 2010; Richer & Beatty, 1985), even if they are not well understood (see below). Further, as mentioned above, the general shape of the pupil data during search could be related to systematic PFE that was not accounted for by the correction process. For these reasons, the overall patterns of dilation during search are not a solid basis for inferring specific cognitive processes and are perhaps best taken only as a general indication of the processing effort or of the level of cognitive engagement required to complete the task. Although there is some suggestion that the number of targets influenced pupil dilation, the whole-trial pupil data most likely represent a composite of various underlying cognitive, motor, reflexive, and PFE components.

The fixation-aligned pupillary response averages provide a more detailed insight into the task-related causes of pupil dilation. The effects can be summarized as follows. In line with Klingner's (2010) original findings, fixations on targets but not distractors reliably evoked small transient pupil dilations (see Figure 11). Refixations, on the other hand, evoked significant pupil modulation for both targets and distractors, and the average waveforms for initial fixations and refixations on both targets and distractors differed significantly (see Figure 14). Finally, pupil modulation following target fixation was not affected significantly by the order in which those targets were discovered in the trial (see Figure 15). The methodological precautions and rigorous criteria for fixation selection justify a cognitive interpretation of the effects associated with fixation and refixation, but what aspect of cognition in particular could be responsible? Using the analogy of processing effort, we might infer that target detection simply required more effort than distractor rejection, or that the task was generally more cognitively demanding in the moments after targets were found. Alternatively, we could suggest that the dilation for targets is associated specifically with an increased memory load, perhaps due to the requirement for encoding target identities and spatial locations in working memory in support of efficient performance. Although plausible, such specific interpretations overlook the possibility that the pupil dilation to targets is more perceptual or reflexive in its nature (e.g. de Gee et al., 2014; Einhäuser et al., 2008; Hupé et al., 2009), or that it originates from cognitive or sensory processes associated with detection, recognition, and decision making (e.g. Einhäuser et al., 2010; Privitera et al., 2010; Wierda et al., 2012). Therefore, before speculating on the precise cognitive origins of the pupil dilation it will help to consider the neuroscience of the underlying mechanism.

As stated at the outset of this communication, a large body of behavioral and neuroscientific research in human and nonhuman animals implicates the activity of the LC-NA system in the cognitive modulation of pupil size. Most notably, microsimulation and neural recording studies in monkeys (Joshi et al., 2016; Rajkowski et al., 1993; Varazzani et al., 2015) have demonstrated a tight temporal coupling between non-luminance mediated pupil-size changes and the moment-to-moment activation of the LC, suggesting that the true value of cognitive pupillometry as a research tool is in its ability to serve as a proxy for the activity of this subcortical brain structure. The LC has long been broadly associated with arousal, cognition, and autonomic function (Hobson, McCarley, & Wyzinski, 1975; Roussel, Buguet, Bobillier, & Jouvet, 1967), but its precise functional role has been the subject of more detailed theoretical speculation over the last 2 decades. For example, it has been theorized that the primary function of the LC may be to do with adaptive gain and optimal performance (Aston-Jones & Cohen, 2005), network reset (Bouret & Sara, 2005), unexpected uncertainty (Yu & Dayan, 2005), and mind-wandering (Mittner, Hawkins, Boekel, & Forstmann, 2016). Although divergent on some key issues, each of these theoretical standpoints draw heavily on the findings from animal studies in which LC activity was measured directly during task performance, and in this respect they agree that the LC has two distinct modes of output, which correspond to different behavioral states. Specifically, it has a phasic mode, which is characterized by short bursts of activation and supports task engagement / exploitation, and a tonic mode, which is characterized by sustained increase in baseline activation and supports disengagement / exploration.

The free-viewing visual search task of the current experiment required participant engagement and the rapid-serial deployment of attention, which is consistent with the phasic mode of LC operation. The instructions of the task were to count and report the number of targets in an efficient manner, which gives target items more relevance than distractors. As such, discovering a target as opposed to a distractor during search might be considered a more “rewarding” experience in that it provides a greater opportunity for task fulfilment. In this context, the overall effect of pupil dilation for targets but not distractors, displayed in Figure 11, might be viewed simply as a reflection of phasic LC activation in response to task relevant stimuli. This interpretation fits well with similar findings from pupillometry studies in humans (Klingner, 2010a; Privitera et al., 2010; Wierda et al., 2012) and also studies in behaving monkeys where phasic LC activity was observed immediately following the detection of target but not distractor stimuli (e.g. Aston-Jones, Rajkowski, Kubiak, & Alexinsky, 1994; Hampson, Opris, & Deadwyler, 2010; Rajkowski, Kubiak, & Aston-Jones, 1994; Usher, Cohen, Servan-Schreiber, Rajkowski, & Aston-Jones, 1999). The function of phasic NA release following target detection could be to facilitate memory consolidation of the targets and their locations, but a more general interpretation that fits with central ideas of the network reset (Bouret & Sara, 2005) and adaptive gain (Aston-Jones & Cohen, 2005) theories of LC-NA function is that it serves to enhance the responsivity of cortical circuits involved in task-specific decision processes (e.g. is that a target or distractor?), thereby facilitating the correct behavioral or cognitive response (e.g. add plus one to the running total of targets, terminate the search). Pursuing this line of thought, the phasic pupil responses observed during visual search are not indicative of specific cognitive processes, such as target detection or memory accumulation; rather they reflect transitions between cognitive states following the outcome of task-related decisions.

In his visual search application of the fixation-aligned pupillary response averaging technique Klingner (2010a) reported that pupil dilation began up to 500 ms before a target was revisited, suggesting that this could be linked to saccade planning to reaffirm the location of a previously visited item. Our effects of refixation did not emerge so early, but there were significant differences between initial fixations and refixations for both targets and distractors. In particular, refixations on targets elicited a larger peak which came around 320 ms earlier than the peak for target discoveries, and rather unexpectedly, a similar component was also present for distractor refixations. Why should refixations on targets and distractors affect the pupil-LC response in this way? One possible explanation is that the refixation component of pupil dilation reflects a higher level cortical mechanism, perhaps originating in the prefrontal and frontoparietal areas (see Peinkhofer et al., 2019), that is associated with an awareness of having already counted a target or rejected a distractor. Such a mechanism would support efficiency by ensuring that already-discovered targets are not added to the running total and time is not wasted by searching previously inspected items. In this sense, the increased pupil dilation following refixations may reflect cortical mechanisms associated with adaptive behavior and maintaining efficient task performance. Such error-related pupil dilation was reported recently in a study by Maier, Ernst, & Steinhauser (2019), for example. It is also possible however that the refixation component seen for both targets and distractors was caused by systematic PFE related to the spatiotemporal distribution of eye movements around refixations, although this was not explored in these data.

The button presses, which terminated searches, in this experiment were associated with significant pupil dilations. Similar effects of button pressing have been observed throughout the pupillometry literature in a variety of tasks (e.g. de Gee et al., 2014; Einhäuser et al., 2010; Einhäuser et al., 2008; Fahle, Stemmler, & Spang, 2011; Hupé et al., 2009; Kloosterman et al., 2015; Porter et al., 2007; Privitera et al., 2010; Richer, Silverman, & Beatty, 1983; Smallwood et al., 2011) including one which simply involved pressing a button at paced intervals in the absence of any particular task instructions or stimulation (Richer & Beatty, 1985). The indication is that phasic LC activation is involved with motor preparation and execution, perhaps to facilitate the consolidation of task-related decision processes. However, it seems that the largest contributing factor to the pupillometric effect of button pressing is not the decision process, but rather the motor act itself. When task-related decisions are made covertly and indicated with a button press at a later time, as in this experiment and in Einhäuser et al. (2010), the corresponding pupil dilations are much smaller than those which are seen at the actual time of button pressing. The neuroscience is not clear on why this might be the case, but Varazzani et al.'s (2015) observations in primates offer a potential explanation. The primates in question were trained to perform a reward / effort task, which required them to squeeze a bar with an amount of force indicated by a cue at the start of the trial. During this task, the firing rate of LC neurons around the time of action was highly correlated with pupil diameter following overt responses as well as the force with which those responses were made. This was the case even after removing the impact of task parameters, which implies that the motor component of the pupil-LC response exists over and above the requirements of the task. Varazzani et al. suggested that the phasic LC activation coextensive with pupil dilation and response force may be serving to mobilize both the physical (muscular) and physiological (autonomic) energy required to complete the action. Such an interpretation fits well with one previous pupillometry study in humans where pupil dilation scaled with the required actuation force of a button response (Richer & Beatty, 1985), and it is generally consistent with research and theory implicating autonomic nervous activity in both physiological (Acevedo et al., 2007; Collet, Roure, Rada, Dittmar, & Vernet-Maury, 1996) and cognitive effort (Howells, Stein, & Russell, 2010; Kahneman & Beatty, 1966; Webb et al., 2010).

## Conclusion

We used a robust synthesis of experimental methods and averaging techniques to gain insight into the cognitive pupil dynamics of free-viewing visual search. Data-driven correction of the PFE was successfully applied but had little effect on statistical outcomes or the appearance of the data, suggesting that rendering eye movements (mostly) random through task and stimulus design may be a sufficient countermeasure. Whole-trial analysis of the pupil data shed light on the general pattern of pupil modulation during search and indicated that pupil size was greatest on average for trials with three targets. The fixation-aligned pupillary response averages revealed transient pupil modulations following initial fixations on targets but not distractors, and refixations on both targets and distractors. Considered in light of the behavioral context and the known functional association between the pupil and the LC-NA system, these phasic dilations may reflect coextensive NA release, which serves to facilitate behavioral or cognitive responses to the outcome of task-related decision processes. Further developments in the neuroscience of cognitive pupillometry will test this interpretation and shed new light on these data.
